# The Opposite Effect of c-Jun Transcription Factor on Apolipoprotein E Gene Regulation in Hepatocytes and Macrophages

**DOI:** 10.3390/ijms20061471

**Published:** 2019-03-23

**Authors:** Violeta G. Trusca, Elena V. Fuior, Dimitris Kardassis, Maya Simionescu, Anca V. Gafencu

**Affiliations:** 1 Institute of Cellular Biology and Pathology “N. Simionescu”, 050568 Bucharest, Romania; violeta.trusca@icbp.ro (V.G.T.); elena.fuior@icbp.ro (E.V.F.); maya.simionescu@icbp.ro (M.S.); 2 Laboratory of Biochemistry, University of Crete Medical School and Institute of Molecular Biology and Biotechnology, Foundation for Research and Technology of Hellas, Heraklion, 71003 Crete, Greece; kardasis@imbb.forth.gr

**Keywords:** apoE, apolipoproteins, c-Jun, AP-1, promoter, enhancer, ME.2, hepatocytes, macrophages

## Abstract

Apolipoprotein E (apoE) is mainly secreted by hepatocytes and incorporated into most plasma lipoproteins. Macrophages, which accumulate cholesterol and are critical for the development of the atherosclerotic plaque, are also an important, albeit smaller, apoE source. Distal regulatory elements control cell-specific activity of the apoE promoter: multienhancers (ME.1/2) in macrophages and hepatic control regions (HCR-1/2) in hepatocytes. A member of AP-1 cell growth regulator, c-Jun regulates the transcription of various apolipoproteins and proinflammatory molecules implicated in atherosclerosis. We aimed to investigate the effect of c-Jun on apoE expression in macrophages versus hepatocytes and to reveal the underlying molecular mechanisms. Herein we show that c-Jun had an opposite, cell-specific effect on apoE expression: downregulation in macrophages but upregulation in hepatocytes. Transient transfections using ME.2 deletion mutants and DNA pull-down (DNAP) assays showed that the inhibitory effect of c-Jun on the apoE promoter in macrophages was mediated by a functional c-Jun binding site located at 301/311 on ME.2. In hepatocytes, c-Jun overexpression strongly increased apoE expression, and this effect was due to c-Jun binding at the canonical site located at −94/−84 on the apoE proximal promoter, identified by transient transfections using apoE deletion mutants, DNAP, and chromatin immunoprecipitation assays. Overall, the dual effect of c-Jun on apoE gene expression led to decreased cholesterol efflux in macrophages resident in the atherosclerotic plaque synergized with an increased level of systemic apoE secreted by the liver to exacerbate atherogenesis.

## 1. Introduction

Apolipoprotein E (apoE) is a glycoprotein with a molecular mass of 35 kDa, comprising 299 amino acids [1,2]. It functions as a ligand in receptor-mediated endocytosis of lipoprotein particles, mediating lipid transfer between the circulating lipoproteins and tissues through its functional domains: the lipid-binding region and the receptor-binding region [3,4]. ApoE facilitates the internalization of lipids by hepatic and extrahepatic cells. Besides its atheroprotective effects, apoE has anti-inflammatory, antiproliferative, and immunomodulatory properties [5].

The liver is the main apoE producer (up to 75% of the total amount). Other important local sources are macrophages, astrocytes, adipocytes, the spleen, kidneys, the adrenal gland, testes, and the skin [6]. Secreted by hepatocytes, apoE is incorporated into most lipoprotein types. Systemic apoE deficiency leads to atherosclerosis in humans and in animal models [7,8,9,10]. Studies in transgenic mice have shown that selective apoE expression only in macrophages inhibits atherosclerosis without affecting plasma lipid levels [11]. Macrophage-secreted apoE contributes to reverse cholesterol transport in vivo by redirecting the excess cholesterol produced by peripheral tissues to the liver for removal [12].

In humans, apoE is encoded by a single gene on chromosome 19, consisting of four exons and three introns, with a total length of 3646 bp [13,14]. The *apoE* gene belongs to a gene cluster located in the proximity of the apoC-I, apoC-IV, and apoC-II genes. The liver-specific expression of the human apoE/apoC-I/apoC-IV/apoC-II gene cluster is modulated by two hepatic control regions, HCR-1 and HCR-2 [15,16]. In macrophages, adipocytes, and astrocytes, apoE expression is controlled by two distal multienhancers, ME.1 and ME.2, located 3.3 and 15.9 kb, respectively, downstream of the apoE gene [17]. The regulation of apoE transcription is a cell-specific process and involves the interaction of several transcription factors with the proximal and distal regulatory regions [18]. Previously, we established that apoE gene expression in macrophages is downregulated through a mechanism involving tumor progression locus 2 (Tpl-2) and mitogen-activated protein kinase kinase kinase 1 (MEKK1) signaling molecules that converge to activate nuclear factor kappa B (NF-κB) and activator protein (AP-1) transcription factors, which interact with a noncanonical site located in the apoE core promoter located at position -55/+73 [19]. Metformin, a drug with anti-inflammatory properties, is able to revert the downregulatory effect of endotoxin on apoE expression [20].

The c-Jun transcription factor is a major component of the AP-l complex, which plays a role in growth and differentiation [21]. The AP-1 complex is composed of dimers between the Jun (c-Jun, JunB, and JunD) and Fos family members (c-Fos, FosB, Fra-1, Fra-2) or other basic region-leucine zipper (bZIP)-containing proteins. The dimerization is mediated by their leucine zipper domains, while recognition of the DNA binding site is mediated by the basic regions. A broad spectrum of factors, including growth factors and phorbol myristate acetate (PMA), stimulate c-Jun expression [22]: c-Jun transcription is further stimulated by its own gene product [23]. After c-Jun proteins dimerize with c-Fos or other members of the AP-1 family, the complex binds DNA at the consensus sequence 5’-TGAG/CTCA-3’, found in the promoters or enhancer regions of target genes [24,25]. Phosphorylation by c-Jun N-Terminal Kinase (JNK), mainly at Ser63 and Ser73, may further enhance the regulatory potential of c-Jun [26]. In C57BL/6 mice, adenovirus-mediated gene transfer of this dominant negative mutant (c-Jun DN), lacking a major part of the transactivation domain, had induced hepatic apoE overexpression, which caused dyslipidemia [27].

The aim of this study was to establish the cell-specific molecular mechanisms of the inflammatory transcription factor c-Jun on apoE expression in two key cell types involved in atherosclerosis: hepatocytes (responsible for the systemic apoE level) and macrophages (the local supplier of apoE in the atherosclerotic plaque, but also the precursor of foam cells). We report here the opposite effects of AP-1 factors on the apoE gene promoter in hepatocytes and macrophages, which led to contrary transcriptional regulation: c-Jun overexpression increased apoE expression in hepatocytes but inhibited apoE expression in macrophages. These cell-specific molecular mechanisms of apoE gene regulation took place in different cellular contexts, in the presence of various cofactors that altered the c-Jun effect.

These opposite regulatory mechanisms of apoE in the liver and macrophages under inflammatory conditions may synergize to promote the evolution of atherosclerosis.

## 2. Results

### 2.1. Apolipoprotein E Expression Was Differentially Modulated by AP-1 Transcription Factors in Hepatocytes and in Macrophages

**Figure 1:**
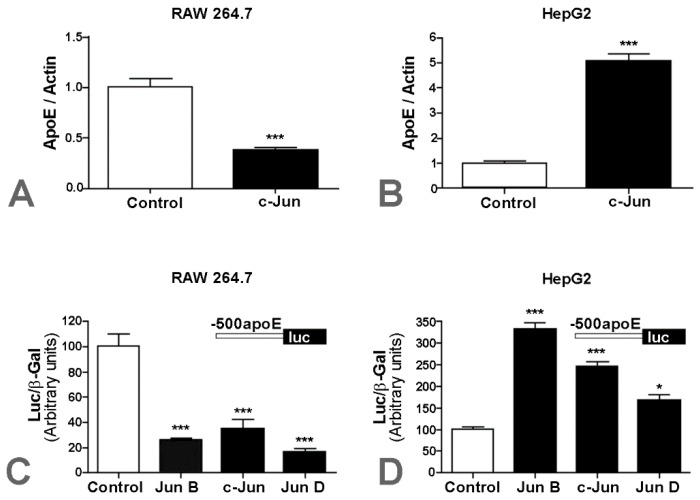
Cell-specific effects of c-Jun on apolipoprotein E (apoE) gene expression in macrophages and hepatocytes. RAW 264.7 (**A**) and HepG2 (**B**) cells were transfected with c-Jun-encoding plasmids, and apoE gene expression was determined by real-time PCR. Overexpression of c-Jun in RAW 264.7 cells caused a reduction in apoE mRNA level down to ~40% of the basal apoE level (*p* < 0.01). By contrast, c-Jun overexpression in HepG2 cells induced a ~5-fold increase in apoE mRNA levels (*p* < 0.001), compared to control cells; RAW 264.7 (**C**) and HepG2 (**D**) cells were transiently transfected with the construct −500apoE in the absence (control) or in the presence of JunB, c-Jun, or JunD expression vectors. In RAW 264.7, cell JunB overexpression reduced apoE promoter activity to ~25% of the control value, c-Jun caused a reduction of activity to ~30% of the control, and JunD reduced its activity to ~20% of the control (*p* < 0.001 for each case). By contrast, the overexpression of JunB in HepG2 cells increased apoE promoter activity (*p* < 0.001) ~3.4-fold, c-Jun increased its activity (*p* < 0.01) ~2.6-fold, and JunD increased apoE promoter activity (*p* < 0.05) 1.6-fold.

First, we assessed apoE gene expression in hepatocytes and macrophages upon overexpression of AP-1 transcription factors. For this, both cell types were transfected with a c-Jun expression vector, and apoE gene expression was evaluated by real-time PCR. The results showed that c-Jun overexpression in RAW 264.7 macrophages caused a reduction in apoE mRNA level down to ~40% of the basal apoE level (*p* < 0.01) compared to control cells (Figure 1A). By contrast, c-Jun overexpression in HepG2 hepatocytes induced a ~5-fold increase in apoE mRNA levels (*p* < 0.001), as illustrated in Figure 1B. These results revealed an opposite modulation of apoE expression by c-Jun in hepatocytes and macrophages.

Next, we tested the effect of other factors from the AP-1 family (c-Jun, JunB, JunD) on apoE gene expression, and questioned whether this could have been due to an effect on the transcriptional regulation of the apoE promoter. For this purpose, cells were transiently transfected with the construct −500apoE-luc in the absence (control) or in the presence of JunB, c-Jun, or JunD expression vectors. After 36 h, apoE promoter activity was measured based on the luciferase reporter gene and normalized to the activity of β-galactosidase, used as an external reference gene. As shown in Figure 1C, in RAW 264.7 macrophages, JunB reduced apoE promoter activity to ~25% of the control value (*p* = 0.0002), c-Jun caused a reduction of the activity to ~40% of the control (*p* = 0.0005), and JunD reduced its activity to ~20% of the control (*p* = 0.0001). By contrast, JunB overexpression in HepG2 hepatocytes increased apoE promoter activity (*p* < 0.0015) ~3.4-fold, c-Jun overexpression increased its activity (*p* < 0.01) ~2.6-fold, and JunD increased apoE promoter activity (*p* < 0.05) 1.6-fold (Figure 1D).

### 2.2. Overexpression of c-Jun Transcription Factors in Macrophages Repressed apoE Promoter Activity, a Process Enhanced by the Multienhancer

**Figure 2:**
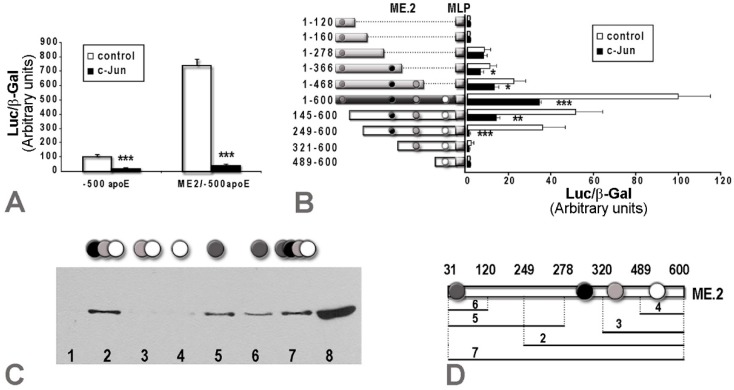
In macrophages, c-Jun bound to ME.2 inhibited apoE promoter activity. (**A**) RAW 264.7 macrophages were transiently transfected with -500apoE construct or ME.2/-500apoE construct in the absence (control) or in the presence of c-Jun expression vectors: c-Jun overexpression in RAW 264.7 cells caused a reduction in the activity of the -500apoE promoter to ~30% of the control (*p* < 0.001), while in the presence of ME.2 (ME.2/-500apoE construct), apoE promoter activity drastically decreased to ~7% of the control (*p* < 0.001); (**B**) RAW 264.7 cells were transfected with a series of plasmids containing various deletion mutants of ME.2 joined to a minimal promoter (MLP) in front of a luciferase gene reporter: c-Jun overexpression significantly decreased the activity of the full-length ME.2 (1–600), as well as of all the mutants that contained a c-Jun binding site located at 301 bp in ME.2 (1–366, 1–468, 145–600, 249–600). By contrast, the fragments containing c-Jun binding sites located at 31, 381, and 495 bp in ME.2 were not affected by c-Jun overexpression (fragments 1–120, 1–160, 1–278, 320–600, 489–600); (**C**) c-Jun binding to the ME.2 region in LPS-treated RAW 264.7 macrophages was tested by DNAP assays using the following fragments of ME.2: 249–600, 320–600, 489–600, 31–278, 31–120, and 31–600. The c-Jun transcription factors bound to the following ME.2 fragments: 249–600 (lane 2), 31–278 (lane 5), 31–120 (lane 6), and 31–600 (lane 7), while the c-Jun factors did not bind to the 320–600 and 489–600 ME.2 fragments (lane 3 and 4, respectively). As a positive control, a nuclear extract from LPS-treated RAW 264.7 cells was used (lane 8). Binding to nonspecific DNA is shown in lane 1; (**D**) Schematic representation of the fragments used in the DNAP assays of panel C and the c-Jun binding sites located in the ME.2 sequence.

Having established that AP-1 transcription factors decreased apoE expression in macrophages, next we searched for the mechanism underlying this transcriptional modulation. Since we previously found that c-Jun decreased apoE proximal promoter activity [19], we examined whether the effect of c-Jun on apoE promoter activity is affected by the distal enhancer of apoE, multienhancer 2 (ME.2). For this purpose, we transiently transfected macrophages with apoE proximal promoter (construct −500apoE) alone or in the presence of ME.2 (construct ME.2/−500apoE) together with c-Jun expression vectors. As illustrated in Figure 2A, c-Jun overexpression in RAW 264.7 macrophages caused a reduction in the activity of the −500apoE promoter down to ~30% of the control value (*p* = 0.0005), while in the presence of ME.2 (ME.2/−500apoE construct), apoE promoter activity was drastically decreased to ~7% of the control (*p* = 0.0007).

To identify the c-Jun binding sites located on ME.2 responsible for apoE promoter downregulation, we searched for the potential active elements using the TRANSFAC database [28], and we found four putative sites starting at positions 31, 301, 381, and 495 in ME.2. Then, we tested the functionality of these sites by transient transfection experiments using full-length ME.2 and 5’- and 3’-deletion mutants of ME.2 cloned in a construct containing a minimal promoter. The data obtained showed that c-Jun overexpression decreased the activity of the full-length ME.2 to 35% of the control value (Figure 2B, 1–600). Using 3’-deletion mutants of ME.2 containing only the first c-Jun binding site (located at 31 bp), no repression of ME.2 by c-Jun was noticed (Figure 2B, ME.2 fragments: 1–120, 1–160, 1–278). By contrast, when the second c-Jun putative binding site (located at 301 bp) was present, a significant repression of ME.2 activity by c-Jun was found (Figure 2B, fragment 1–366). A similar effect was also obtained when the construct containing the 1–468 fragment of ME.2 (Figure 2B, fragment 1–468) was employed. From all the 5’-deletion mutants of ME.2, only the activity of the fragments containing the second c-Jun binding site located at 301 in ME.2 (Figure 2B, fragments 145–600 and 249–600) was significantly repressed by c-Jun, while the fragments containing the third and the fourth c-Jun binding sites (located at positions 381 and 495, respectively, in ME.2) were not affected by c-Jun overexpression (Figure 2B, fragments 321–600 and 489–600, respectively).

To test whether the c-Jun transcription factor binds to the ME.2 region, we performed DNA pull down (DNAP) assays using ME.2 deletion fragments 249–600, 320–600, 489–600, 31–120, 31–278, and 31–600, amplified by PCR using RV3 biotinylated primer, and nuclear extracts obtained from LPS-treated RAW 264.7 macrophages. The results showed that c-Jun transcription factors bonded to the ME.2 fragments 249–600 (Figure 2C, lane 2), 31–278 (Figure 2C, lane 5), 31–120 (Figure 2C, lane 6), and 31–600 (Figure 2C, lane 7), while c-Jun factors did not bind to ME2 fragments 320–600 and 489–600 (Figure 2C, lanes 3 and 4, respectively). A nuclear extract from RAW 264.7 cells incubated 24 h with LPS (1µg/mL) was used as a positive control (Figure 2C, lane 8). Nonspecific DNA was used as a negative control and showed no binding of c-Jun proteins (Figure 2C, lane 1). In conclusion, DNAP experiments revealed that both c-Jun binding sites located at 31 and 301 bp in ME.2 could bind to ME.2, while the other two putative binding sites located at positions 381 and 495 in ME.2 were not effective in c-Jun binding. However, considering the data obtained from the transfection experiments, we can safely assert that only the c-Jun binding site located at 301 bp in ME.2 was functional.

Taken together, the data presented in Figure 2 showed that c-Jun overexpression in macrophages repressed apoE proximal promoter activity, and a further decrease was detected when the apoE promoter was cloned in the presence of ME.2, the distal enhancer of apoE. Thus, c-Jun factors bound not only to the apoE proximal promoter, but also to ME.2 in the region starting at position 301.

### 2.3. In hepatocytes, c-Jun Factors Bound to the apoE Promoter and Upregulated Its Activity

**Figure 3:**
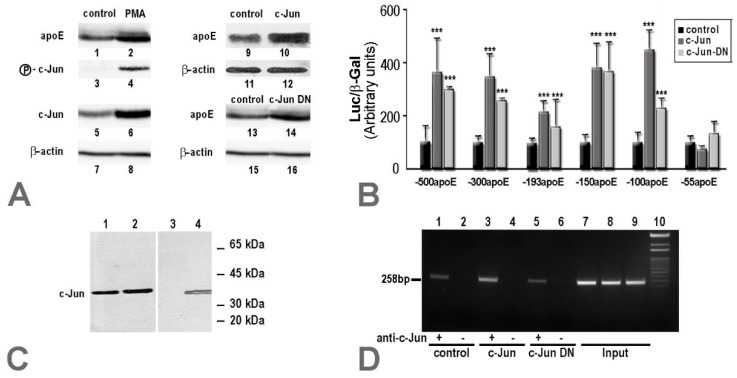
In hepatocytes, c-Jun transcription factors bound to the apoE promoter and upregulated its activity. (**A**) PMA stimulation of HepG2 cells induced phospho-c-Jun expression (lane 4 vs. 3) and increased total c-Jun protein levels (lane 6 vs. 5), which led to an increased apoE protein expression (lane 2 vs. 1), as demonstrated by Western blot. An increase in apoE protein level was noticed also when the cells were transfected to overexpress c-Jun (lane 10 vs. 9). A similar increase in apoE protein level (lane 14 vs. 13) was induced by overexpression of the dominant negative form of c-Jun (c-Jun DN). The expression of β-actin was used as an internal control (lanes 7, 8, 11, 12, 15, and 16); (**B**) From a series of apoE promoter deletion mutants used for HepG2 cell transfection, only the fragment −55apoE was not affected by c-Jun or c-Jun DN overexpression, and the smallest apoE fragment activated was -100apoE; (**C**) DNAP assays using nuclear extracts from HepG2 cells showed that c-Jun proteins bound to −300apoE and −100apoE (lanes 1 and 2, respectively). The positive control represents the c-Jun level in the nuclear extract from HepG2 cells (lane 4). In the negative control, a nonspecific biotinylated DNA fragment was used in the DNAP assays (lane 3); (**D**) Chromatin immunoprecipitation (ChIP) assay using chromatin from HepG2 hepatocytes (control cells or cells overexpressing c-Jun or c-Jun DN) and anti-c-Jun antibodies showed that c-Jun bound to the apoE promoter. Higher amounts of apoE promoter DNA were immunoprecipitated from HepG2 cells overexpressing c-Jun (lane 3) compared to control cells (lane 1) or hepatocytes transfected with c-Jun DN (lane 5). In the negative controls, nonspecific IgG was used to precipitate chromatin from control cells (lane 2), HepG2 cells transfected with c-Jun (lane 4) or c-Jun DN (lane 6), and no PCR products were identified. The input, representing 1% from each type of chromatin sample obtained from the control or c-Jun/c-Jun DN transfected hepatocytes, produced the expected band (lanes 7, 8, and 9, respectively).

Since our data showed that c-Jun enhanced apoE expression in hepatocytes, we questioned whether c-Jun phosphorylation would have been responsible for this. Thus, we evaluated apoE expression in hepatocytes, where c-Jun phosphorylation was induced by PMA. For this, HepG2 hepatocytes were treated with 100 nM of PMA for 48 h, and cellular apoE protein levels were measured by immunoblotting. The results showed that PMA stimulation induced phospho-c-Jun expression (Figure 3A, lane 4 vs. 3) and increased total c-Jun protein levels (Figure 3A, lane 6 vs. 5), which led to increased apoE protein expression (Figure 3A, lane 2 vs. 1). The same upregulatory effect on apoE expression at the protein level was detected also when the cells were transfected to overexpress c-Jun and analyzed by immunoblotting (Figure 3A, lane 10 vs. 9). Surprisingly, the same increase in apoE protein level (Figure 3A, lane 14 vs. 13) was induced by overexpression of c-Jun DN protein, which lacks a major part of the transactivation domain (amino acids 3–122). In all the experiments, the expression of β-actin was used as an internal control (Figure 3A, lanes 7, 8, 11, 12, 15, and 16).

Since we determined the minimal region responsible for the repression of the apoE gene in macrophages to be located in the (−55/+73) region of the apoE promoter [19], we further investigated what is the minimal region of the apoE proximal promoter that is upregulated by c-Jun or c-Jun DN in hepatocytes. For this, a series of apoE promoter deletion mutants (−500apoE, −300apoE, −193apoE, −150apoE, −100apoE, and −55apoE) were used to transiently transfect HepG2 cells in the absence or in the presence of c-Jun factors. All of the apoE promoter fragments tested except −55/+73 were activated by c-Jun or c-Jun DN overexpression (Figure 3B). The smallest apoE promoter fragment whose transcriptional activity was enhanced by c-Jun overexpression was −100/+73, in accordance with TRANSFAC analysis [28], which predicted a c-Jun binding site in the region −94/−84 of the apoE promoter. 

To provide evidence for c-Jun transcription factors binding to the apoE promoter in hepatocytes, we performed complementary DNAP and ChIP assays. In vitro c-Jun binding to biotinylated apoE promoter fragments was tested with DNAP assays, using nuclear extracts obtained from HepG2 hepatocytes. These experiments showed that c-Jun proteins bound to both apoE promoter fragments tested, namely −300apoE and −100apoE (Figure 3C, lanes 1 and 2, respectively). As a positive control, the c-Jun level in the nuclear extract of HepG2 hepatocytes was assessed (Figure 3C, lane 4), and as a negative control, a nonspecific biotinylated DNA fragment was used in the DNAP assays (Figure 3C, lane 3). Next, we tested whether c-Jun binding sites on the apoE promoter are functional in hepatocytes through ChIP experiments. For this purpose, cross-linked chromatin from HepG2 hepatocytes (control cells or cells transiently transfected with c-Jun or c-Jun DN) was immunoprecipitated using anti-c-Jun antibodies, and the DNA sequence of the apoE promoter was identified by PCR performed using primers for the region −254/+4 of the apoE gene. The results of the ChIP experiments indicated that c-Jun transcription factors bound to the apoE promoter in all samples obtained from the control cells or cells transiently transfected with c-Jun or c-Jun DN. However, higher amounts of apoE promoter DNA were immunoprecipitated from HepG2 cells transfected with c-Jun compared to nontransfected cells or cells transfected with c-Jun DN (Figure 3D, lanes 3, 1, and 5, respectively). As negative control, non-specific IgG was used to immunoprecipitate chromatin from control cells or hepatocytes overexpressing c-Jun or c-Jun DN, and no PCR products were identified (Figure 3D, lanes 2, 4, and 6, respectively). The input, representing 1% of the starting sheared chromatin obtained from untreated or hepatocytes transfected with c-Jun or c-Jun DN, produced the expected band (Figure 3D, lanes 7, 8, and 9, respectively). 

In conclusion, the data obtained from the transfection experiments, DNAP and ChIP assays confirmed that the c-Jun binding site located on the apoE promoter at −94/−84 was functional and induced upregulation of the apoE gene in hepatocytes.

## 3. Discussion

Apolipoprotein E (apoE), a protein involved in lipid metabolism, plays an important role in lipoprotein clearance and cholesterol redistribution [2]. This function is accomplished mainly by the apoE secreted by the liver, the organ that provides the required amount of apoE and other apolipoproteins in the plasma. Smaller, but nonetheless important, apoE sources are macrophages [6]. Macrophages are attracted into the atherosclerotic plaque, where they contribute to cholesterol clearance and secrete anti-atherosclerotic and anti-inflammatory factors to counteract atheroma formation. In addition, apoE reduces inflammation, playing a role in the inhibition of lymphocytes and vascular smooth muscle cell proliferation [29,30] and in the conversion of the macrophage phenotype from pro-inflammatory M1 into anti-inflammatory M2 cells [31]. However, macrophages trapped in atherosclerotic lesions under inflammatory stress factors secrete lower amounts of apoE, thus being unable to maintain the homeostasis of the vascular wall. In a previous study, we showed that inflammatory conditions downregulate apoE expression in macrophages by a process mediated by inflammatory transcription factors such as AP-1 and NF-κB [19].

Here, we report that c-Jun had a dual effect on apoE expression depending on the cell type: in macrophages, c-Jun decreased apoE expression, while in hepatocytes c-Jun increased apoE expression (Figure 1). A cell-specific modulation of apoE was also recorded for glucocorticoids, which increased apoE expression in macrophages, without any significant interference on the hepatic apoE level [32].

Prompted by the opposite effects of c-Jun on the apoE gene, we searched for the molecular mechanisms of this regulation in hepatocytes and macrophages. Our experiments demonstrated that this alteration in apoE expression was a transcriptional cell-specific modulatory mechanism. A similar cell-specific modulatory effect of c-Jun on apoE promoter activity was obtained by overexpression of JunB and JunD, two other members of the AP-1 family (Figure 1C,D). 

**Figure 4:**
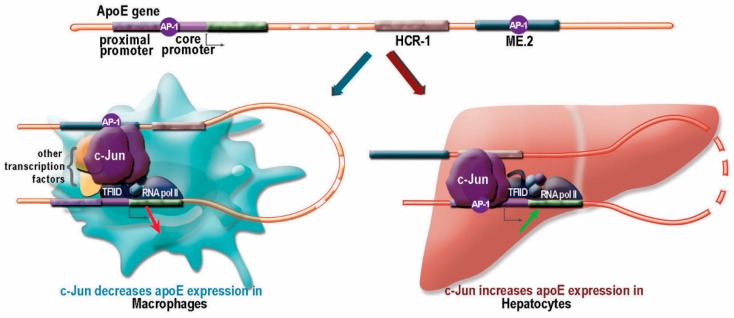
Schematic representation of cell-specific effects of c-Jun on apoE gene expression. In macrophages, c-Jun binding to the apoE core promoter downregulated apoE expression, in conjunction with other transcription factors. ME.2 distal regulatory element was brought into the proximity of the apoE promoter, and the functional AP-1 binding site on ME.2 additionally potentiated c-Jun repression of apoE (left side). In hepatocytes, the HCR-1 distal regulatory element interacted with the apoE promoter, but apoE expression was upregulated by c-Jun in a classical manner through binding to a canonical binding site located at −94/−84 in the apoE proximal promoter, (right side). AP-1 sites are illustrated as purple circles.

Our data showed that in macrophages, due to an interaction with the distal regulatory element ME.2, apoE expression specifically increased 7–10 fold (Figure 2A, controls). We have previously inferred that the apoE core promoter is involved in the c-Jun downregulatory effect by indirect interactions, since no consensus binding sites were detected in the region −55/+73 of the apoE promoter [19]. We searched for the possible influence of c-Jun binding sites on the ME.2 distal regulatory element. The data presented in Figure 2A shows that the c-Jun downregulatory effect on the apoE promoter was more robust in the presence of ME.2 (decrease to 7%) compared to the downregulation of the promoter activity alone (decrease to 30%). Thus, we further investigated the influence of the c-Jun binding sites located on ME.2 on apoE promoter activity. A DNAP assay using a nuclear extract from LPS-treated macrophages as well as transient transfection experiments in RAW 264.7 cells using fragments of ME.2 joined to a minimal promoter in front of the luciferase reporter gene showed a functional c-Jun binding site located on ME.2 in the region starting with 301 bp (Figure 2). The proposed mechanism of apoE downregulation in macrophages is illustrated in Figure 4.

In hepatocytes, c-Jun overexpression induced a strong increase in apoE expression. We demonstrated that this upregulation was related to c-Jun binding to the AP-1 binding site located on the apoE proximal promoter (−94 → −84), as illustrated in Figure 3. Surprisingly, this enhancement of promoter activity was independent of c-Jun transactivation, since a deletion mutant of c-Jun (c-Jun DN) missing the transactivation domain of the molecule was able to induce a similar increase in apoE expression. These results could explain the data obtained in vivo in C57BL/6 mice transduced with a recombinant adenovirus expressing c-Jun DN, in which apoE overexpression was induced, triggering dyslipidemia [27]. In hepatocytes, the multienhancers are inactive, but the hepatic control regions 1 and 2 represent the distal regulatory elements that modulate apoE promoter activity. HCR-1 is located in close proximity to ME.2 (at a distance of 0.5 kb). In silico analysis performed with TF Bind (http://tfbind.hgc.jp/) showed that HCR-1, as well as HCR-2, contained eight putative binding sites for c-Jun. It is possible that these c-Jun binding sites further enhance the upregulatory effect of c-Jun on apoE expression in hepatocytes, as illustrated in Figure 4, but this hypothesis needs further investigation. The c-Jun transcription factor is a regulator of hepatocyte proliferation in liver regeneration [33], and it can also modulate the expression of liver-secreted plasma proteins. In contrast to apoE, data from the literature have shown a downregulatory effect of c-Jun on the expression of several apolipoproteins. The treatment of HepG2 cells with the specific JNK inhibitor SP600125 increased apoA-I promoter activity and reduced the level of inhibition of apoA-I promoter by TNFα [34]. Other data have revealed that Jun and ATF-2 factors repressed apoC-III promoter activity in HepG2 cells [35]. Overexpression of c-Jun or JunB proteins has inhibited apoM gene transcription in hepatic cells by competing with HNF-1a for binding to the same regulatory element [36].

In conclusion, the key findings of our study are that c-Jun differentially modulated apoE gene expression in hepatocytes and macrophages by a process schematically represented in Figure 4. The repression of the apoE gene in the macrophages residing in the atherosclerotic plaque may diminish cholesterol efflux and, in conjunction with the enhancement of apoE expression in the dyslipidemic liver, could exacerbate the evolution of atherosclerotic plaque.

Understanding the dual mechanism of apoE gene regulation may facilitate the development of novel therapies. A perspective on atherosclerosis treatment is the design of drugs that counterbalance the effect of inflammatory transcription factors that regulate a series of genes encoding proteins involved in lipid metabolism.

## 4. Materials and Methods

### 4.1. Chemicals

Luciferase assay system, *o*-nitrophenyl β-d-galactopyranoside, and restriction and modification enzymes (T4 DNA ligase, Pfu DNA Polymerase, MMLV reverse transcriptase, calf intestinal alkaline phosphatase) were purchased from Promega Corp. (Madison, WI, USA). FastDigest restriction enzymes were from Thermo Scientific. DMEM with 1‰ or 4.5‰ glucose and fetal calf serum was from EuroClone (Milano, Italy), and the Super Signal West Pico chemiluminescent substrate was from Pierce (Rockford, IL, USA). TRIzol reagent was purchased from Invitrogen Life Technologies (Carlsbad, CA, USA), and Dynabeads M-280 streptavidin magnetic beads were from Invitrogen Dynal (Oslo, Norway). Primers were from Microsynth AG (Balgach, Switzerland). Midori Green Advanced DNA Stain (MG-02) was from Nippon Genetics Europe (Dueren, Germany). Antibodies were from Santa Cruz Biotechnology (Santa Cruz, CA, USA). All other chemicals were from Sigma-Aldrich (St. Louis, MO, USA).

### 4.2. Plasmid Constructions

The apoE proximal promoter (−500apoE) and its fragments ((−300apoE), (−150apoE), (−100apoE), and (−55apoE)) were cloned in a Sac I/Kpn I site in a pGL3 basic vector (Promega) that contained as a reporter the promoterless luciferase (luc) gene, as previously shown [19]. The deletion mutants of ME.2 were described in References [19,37].

### 4.3. Cell Culture

RAW 264.7 mouse macrophages were cultured in DMEM with 1‰ glucose supplemented with 10% fetal bovine serum. HepG2 hepatocytes were grown in DMEM with 4.5‰ glucose and 10% fetal bovine serum.

### 4.4. Cell Transfection

RAW 264.7 macrophages and HepG2 hepatocytes were transfected with c-Jun expression vector by the Ca_3_(PO_4_)_2_ precipitation method. Transfected RAW 264.7 cells were selected with G418 to obtain stably transfected cells overexpressing c-Jun. For HepG2 cells, transfection efficiency was more than 80%, and thus the cells were directly used in the experiments to determine gene expression. To evaluate apoE promoter activity, both cell types were transiently transfected with plasmids containing apoE proximal promoter (−500apoE) or its deletion fragments in pGL3 vector in the presence of expression vectors for c-Jun/c-Jun DN. Luciferase activity was assayed with the Luciferase Assay Kit, and transfection efficiency was normalized to β-galactosidase activity. Each transfection experiment was done in triplicate and repeated at least three times.

### 4.5. Real-Time PCR

Total cellular RNA was extracted using TRIzol reagent, and reverse transcription was performed with a High-Capacity cDNA reverse transcription kit (Applied Biosystems, Waltham, MA, USA). Real-time PCR was performed using TaqMan probes and specific primers for apoE (Hs00171168_m1, Mm01307193_g1) and β-actin genes (Hs01060665_g1, Mm00607939_s1) in duplex reaction with Universal Master Mix on a 7900 HT Applied Biosystems machine (Applied Biosystems). ApoE levels were normalized to β-actin expression.

### 4.6. DNA Pull-Down Assays

This assay was carried out using nuclear extracts from RAW 264.7 cells treated with 1 µg/mL LPS for 24 h or from HepG2 hepatocytes. Biotinylated DNA (corresponding to ME.2 fragments (249–600), (320–600), (489–600)], (31–278), (31–320), (31–600)] or corresponding to the fragments (−100/+4), (−300/+4) of the apoE promoter) were immobilized on Dynabeads M-280 Streptavidin, as described previously [19]. The complexes were washed and then subjected to SDS-PAGE followed by Western blotting using anti-c-Jun antibodies followed by HRP-labeled secondary antibodies. The proteins were revealed using Super Signal West Pico chemiluminescent substrate.

### 4.7. Immunoblotting

HepG2 cells (treated with 100 nM of PMA for 24 h or transiently transfected with c-Jun or c-Jun DN) were washed with phosphate-buffered saline, harvested, and solubilized in Laemmli buffer. The protein samples were subjected to SDS-PAGE and transferred onto nitrocellulose, and the blots were probed with anti-apoE, anti-c-Jun, anti-phospho-c-Jun, and anti-β-actin antibodies followed by appropriate HRP-labeled secondary antibodies.

### 4.8. Chromatin Immunoprecipitation

Chromatin immunoprecipitation experiments were done as previously reported [38] using chromatin from HepG2 hepatocytes (control or overexpressing c-Jun or c-Jun DN). Chromatin was immunoprecipitated with anti-c-Jun antibodies for 18 h at 4 °C and analyzed by PCR using the following primers: Forward -254apoE: 5’-TCCACGCTTGGCCCCC and reverse +4apoE: 5’-TGGGGCTGAGTAGGAC. PCR products were analyzed by agarose gel electrophoresis after staining with Midori Green Advanced DNA Stain.

### 4.9. Statistics

Results were expressed as means ± standard deviation. Statistical significance was calculated using a two-tailed *t*-test (GraphPad Software, San Diego, USA). At the 0.05 level, the population means were significantly different.

## Abbreviations

apoE	Apolipoprotein E
AP-1	Activator protein 1
bp	Base pair
ChIP	Chromatin immunoprecipitation
DNAP	DNA pull-down assays
HCR	Hepatic control region
HRP	Horse radish peroxidase
LPS	Lipopolysaccharide
ME	Multienhancer
MLP	Minimal late promoter
PMA	Phorbol 12-myristate-13-acetate
SDS-PAGE	Sodium dodecyl sulfate polyacryl amide gel electrophoresis
